# The HIDDEN Protocol: An Australian Prospective Cohort Study to Determine the Utility of Whole Genome Sequencing in Kidney Failure of Unknown Aetiology

**DOI:** 10.3389/fmed.2022.891223

**Published:** 2022-05-26

**Authors:** Jacqueline Soraru, Sadia Jahan, Catherine Quinlan, Cas Simons, Louise Wardrop, Rosie O’Shea, Alasdair Wood, Amali Mallawaarachchi, Chirag Patel, Zornitza Stark, Andrew John Mallett

**Affiliations:** ^1^Department of Nephrology and Hypertension, Perth Children’s Hospital, Perth, WA, Australia; ^2^Kidney Health Service, Royal Brisbane and Women’s Hospital, Brisbane, QLD, Australia; ^3^Faculty of Medicine, Institute for Molecular Bioscience, The University of Queensland, Brisbane, QLD, Australia; ^4^Australian Genomics, Murdoch Children’s Research Institute, Melbourne, VIC, Australia; ^5^Royal Children’s Hospital, Melbourne, VIC, Australia; ^6^Murdoch Children’s Research Institute, Melbourne, VIC, Australia; ^7^Department of Paediatrics, University of Melbourne, Melbourne, VIC, Australia; ^8^Department of Medical Genomics, Royal Prince Alfred Hospital, Camperdown, NSW, Australia; ^9^Garvan Institute of Medical Research, Sydney, NSW, Australia; ^10^Genetic Health Queensland, Royal Brisbane and Women’s Hospital, Brisbane, QLD, Australia; ^11^Townsville University Hospital, Townsville, QLD, Australia; ^12^College of Medicine and Dentistry, James Cook University, Townsville, QLD, Australia

**Keywords:** kidney failure, genomics, genetic condition, unknown aetiology, genetic kidney disease

## Abstract

Early identification of genetic kidney disease allows personalised management, clarification of risk for relatives, and guidance for family planning. Genetic disease is underdiagnosed, and recognition of genetic disease is particularly challenging in patients with kidney failure without distinguishing diagnostic features. To address this challenge, the primary aim of this study is to determine the proportion of genetic diagnoses amongst patients with kidney failure of unknown aetiology, using whole genome sequencing (WGS). A cohort of up to 100 Australian patients with kidney failure of unknown aetiology, with onset <50 years old and approved by a panel of study investigators will be recruited *via* 18 centres nationally. Clinically accredited WGS will be undertaken with analysis targeted to a priority list of ∼388 genes associated with genetic kidney disease. The primary outcome will be the proportion of patients who receive a molecular diagnosis (diagnostic rate) *via* WGS compared with usual -care (no further diagnostic investigation). Participant surveys will be undertaken at consent, after test result return and 1 year subsequently. Where there is no or an uncertain diagnosis, future research genomics will be considered to identify candidate genes and new pathogenic variants in known genes. All results will be relayed to participants *via* the recruiting clinician and/or kidney genetics clinic. The study is ethically approved (HREC/16/MH/251) with local site governance approvals in place. The future results of this study will be disseminated and inform practical understanding of the potential monogenic contribution to kidney failure of unknown aetiology. These findings are anticipated to impact clinical practice and healthcare policy.

**Study Registration:**

[https://dora.health.qld.gov.au], identifier [HREC/16/MH/251].

## Background

An estimated 10% of chronic kidney disease (CKD), including kidney failure (KF), is attributed to an underlying genetic condition (1, 2). Identifying a genetic cause can aid medical management (3), with potential benefits in slowing the rate of kidney function decline, prognosticating risk of disease recurrence in the event of kidney transplantation, improving monitoring or management of extra-renal manifestations, impacting informed reproductive decisions, and facilitating cascade testing (3). Many causes of CKD lead to KF with the prevalence of KF in 2020 being almost 32,000 individuals in Australia and New Zealand combined (4). Kidney replacement therapies for KF are resource-intensive [>$80,000/year/patient (5), >1,000,000 admissions/year in Australia (6)] and place a significant burden on individuals, families, and healthcare systems (6).

Up to 5% of Australian and 4% of New Zealand KF populations do not have a diagnosis for their kidney disease (6). Many diseases that cause KF have genetic origins; however, if there are no clear clinical features to indicate genetic disease, or patients present in later-stage disease, diagnosis can be delayed or missed. Missed or delayed diagnosis potentially affects disease progression and time to KF (3). Genetic diagnosis also significantly impacts kidney transplantation, with between 20 and 51% of transplant recipients under the age of 50 having a monogenic cause for their kidney disease (7, 8). Early molecular diagnosis can clarify the risk of primary disease recurrence in kidney transplant recipients, limiting the use of therapies with significant side effect profiles and helping to identify potential unaffected living-related kidney donors (3).

The benefits of detection of genetic kidney disease are not limited to the individual. Genetic diagnosis also facilitates the early identification of affected relatives which could vastly improve their medical management outcomes, including their trajectory to KF (9). For this reason, there is an increasing emphasis on integrating genomics into mainstream healthcare (10), including kidney medicine. The KidGen Collaborative is an Australian consortium of nephrology and genetics clinicians, diagnostic laboratory scientists and researchers committed to improving diagnosis and treatment of genetic kidney disease (11). It utilises a network of multidisciplinary clinics across Australia, along with a research pipeline to identify and functionally characterise novel disease-causing genes in families with no diagnosis. A previous multisite Australian prospective cohort study of 204 kidney disease patients with a suspected monogenic cause revealed a diagnostic yield from exome sequencing of 39% (12).

The aim of the HIDDEN (wHole genome Investigation to iDentify unDEtected Nephropathies) study is to investigate the utility and diagnostic yield of clinically accredited whole genome sequencing (WGS) in patients with early-onset unexplained KF, in order to inform how genomic testing should be translated into the clinical care of this patient group. The results will provide “real world” applicability of WGS in the kidney clinic. In addition, the study is anticipated to improve our understanding of genotype-phenotype correlations and potentially lead to the discovery of new genes and pathogenic variants in kidney disease.

## Methods and Design

### Aims

The primary aim of the study is to compare standard-of-care (no further diagnostic investigation) against WGS for younger patients with kidney failure of unknown cause. The primary outcome is to:

1. Determine if WGS can identify a genetic aetiology for a prospectively recruited cohort of Australian patients with KF of unknown cause.

### Recruitment of Patients

**FIGURE 1:**
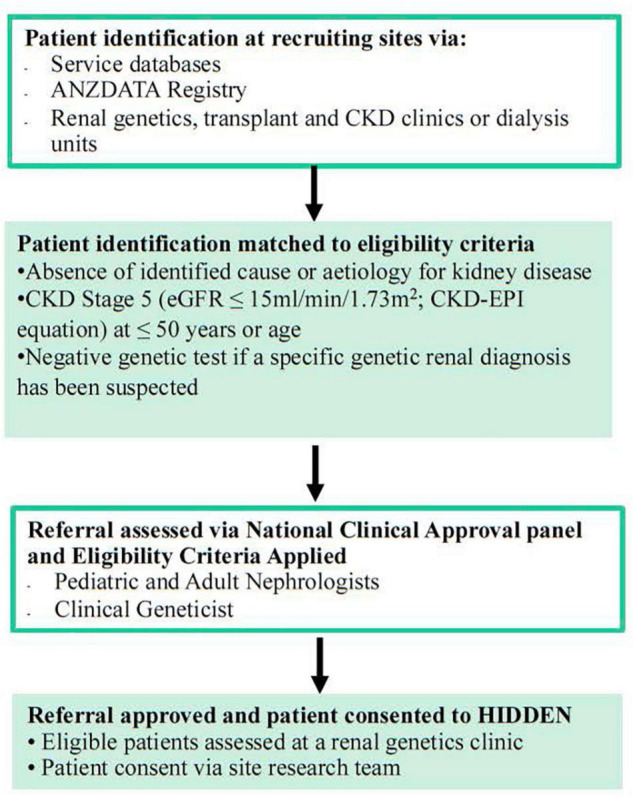
Recruitment flow into HIDDEN.

A cohort of up to 100 Australian patients with KF with no definitive diagnosis and matching the inclusion criteria of the following (Supplementary Material: S5 File); an absence of identified cause or aetiology for kidney disease, CKD Stage 5 (eGFR ≤ 15 ml/min/1.73 m2; CKD-EPI equation) at ≤50 years of age and negative genetic test if a specific genetic kidney diagnosis has been suspected will be recruited into the study. The exclusion criteria include those with an existing kidney clinical or phenotypic diagnosis, specifically; a likely or proven diabetic nephropathy, renovascular disease, renal sarcoidosis, primary nephrotic-range proteinuric disorder, or tuberculosis, paraproteinemia (except when excluded on kidney biopsy), exposure to nephrotoxin causing kidney dysfunction, or obstructive uropathy, nephromegaly (>14 cms for adults; normagram for paediatric patients) and a family history of cystic kidneys, identified glomerular disorder on kidney biopsy that clarifies a diagnosis, identified and proven primary renal diagnosis (as per ANZDATA coding), and isolated Congenital Anomaly of Kidney and Urinary Tract (CAKUT). The criteria for CAKUT exclusion will be based upon clinical consensus of the National Panel, with classification of CAKUT approximating that of Figure 1 from Khan et al. (13). Cases with a lower yield of defects in urinary system morphogenesis ranging from the number, structure, and/or position of the kidneys; obstructive or non-obstructive dilatation of the urinary tract; to dysplastic kidney lesions, including cystic disorders will be evaluated to allow for appropriate exclusion. Those excluded from the study will continue with standard clinically indicated care. The recruitment pathway will involve the 18 local sites nationally identifying potential participants (Figure 1). The recruitment pathway will involve the local sites identifying candidate participants believed to meet the inclusion/exclusion criteria *via* local service databases and local site ANZDATA registry reports/searches. Sites may reach out to other services that are within their local, regional, or state-wide referral networks through standard or telehealth means to identify additional individuals for participation. Each potential participant will then be nominated by their local site clinician for assessment of potential suitability by a Panel of the study’s lead clinical investigators (AJM, CP, ZS, CQ, and ACM) through a nomination form (Supplementary Material: S1 File). The Consensus Panel will apply the inclusion/exclusion criteria and decision on suitability to recruit will be reached within 1 week of nomination and on the basis of a majority vote with the outcome communicated to the nominating clinician by the Panel Chair (AJM). This iterative case identification, nomination and review process will ensure consistency and adherence to the inclusion criteria. For more detailed information on the recruitment pathway refer to Supplementary Figure 1.

### Prospective Cohort Data Collection and Analysis

Australia wide there are 1,150 eligible patients and a timeframe of 1–2 years for identifying, consenting, enrolling, and sequencing of 100 cases is anticipated nationally. A consecutively identified series of eligible KF patients will be recruited through their standard of care (SOC) where regular blood testing occurs and sampling for DNA extraction/storage can occur during SOC pathways. The turnaround time from consent to sample receipt to results will be 3 months to inform patient care. In addition, parental and familial samples will be collected if required for variant assessment such as for phasing or segregation. Consent for recruitment and participation will be obtained from individual recruitment sites (Supplementary Figure 1 and Supplementary Material: S2 File). The prospective cohort will be compared with a retrospective cohort of matched controls from the ANZDATA registry (the national dialysis and kidney transplantation data), in whom WGS has not been applied and an unresolved diagnostic odyssey exists. The registry-coded Primary Renal Diagnosis is “Unknown” and outcomes are reported by ANZDATA in their public annual reporting. A pilot sample of ten initial HIDDEN recruits from one recruiting state (Queensland) will have pharmacogenomic testing as a proof of principle.

Detailed data collection on phenotype will be entered for each consented participant into a secure REDCap database (Supplementary Material: S3 File). WGS analysis will be undertaken in facilities with NATA (The National Association of Testing Authorities) accreditation for clinical reporting. This includes specific NATA accreditation to perform WGS in accordance with the requirements of the National Pathology Accreditation Advisory Council of Australia and AS ISO 15189-2013. A gene panel comprising ∼388 genes associated with kidney disease (KidneyOme) will be applied in the analysis. Previous work (12) informed the curated list of genes for inclusion in the KidneyOme and a final gene list is available in Supplementary Material: S4 File and was informed by a virtual gene panel that is clinically available.^1^ WGS is optimal at identifying a range of variants, including copy number variants (CNVs; not uncommon in inherited kidney disease), splicing, and intronic variants. The diagnostic laboratory WGS method is being provided by three NATA accredited laboratories providing a mean coverage of >30X, with >98% of canonical protein coding transcripts and splice sites covered at >15X. Sensitivity is >99% for single nucleotide variants and >95% for small indels <20 bp. Sensitivity is >81% for copy number losses <500 bpin size and 96% for >500 bp.

Genome analysis in those clinically accredited diagnostic laboratories is being undertaken with the Illumina sequencing genome platform with reads being aligned to the human genome reference sequence (GRCh37/GRCh38) and variant calls made using the Genome Analysis Tool Kit and ClinSV. Single nucleotide and small indel variants will be classified according to the joint consensus recommendations of the American College of Medical Genetics and Genomics and the Association for Molecular Pathology. These will be reported using HGVS nomenclature. Copy number variants will be classified similarly according to recommendations of the American College of Medical Genetics and Genomics.

**FIGURE 2:**
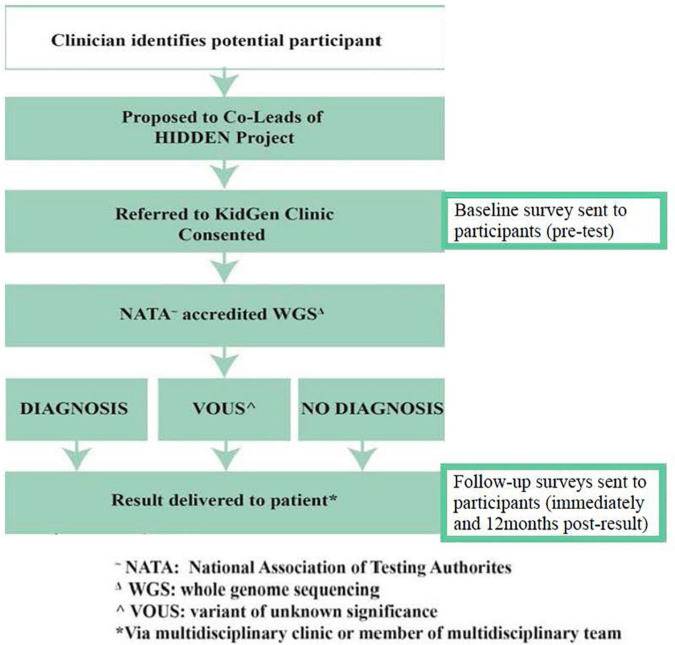
Data collection and WGS workflow for HIDDEN.

Where there is no diagnosis, or the diagnosis is uncertain, further research analysis will be considered through the KidGen research and functional genomics pipeline to identify candidate genes and new pathogenic variants in known genes (Figure 2). The timeframe for recruitment and sequencing is over a 1–2-year period. An electronically communicated REDCap participant survey will be distributed at consent, at or shortly after test return, and 1 year subsequent to that.

### Delivery of Results

All results, where a WGS diagnosis is made or there is an uncertain or uninformative result, will be returned to patients *via* their clinicians at the recruitment sites (Figure 2). Additional input and support from the relevant local KidGen multidisciplinary kidney genetics clinic will be provided if/as required in addition to discussion of any results at the discretion of the recruiting clinician/s at a KidGen National Multidisciplinary Team meeting to ensure closure of the clinic-research-clinic translational loop.

### Ethics Approval

This study has been approved by the Melbourne Health Human Research Ethics Committee (HREC/16/MH/251) *via* approved amendment on 26 February, 2018 (Australian Genomics Health Alliance: Preparing Australia for Genomic Medicine, Protocol V7). All sites across all Australian states and territories achieved subsequent local research governance site specific approval under the National Ethics Statement and associated frameworks of the National Health and Medical Research Council.

## Discussion

The HIDDEN protocol presents the first prospective application of WGS-clinic-based testing in KF patients from all states and territories in Australia. The prospective, multi-site study design along with consistent application of inclusion criteria through a national clinical referral approval panel enables strong consistency and applicability. Previous studies in KF cohorts applied genomic testing in single centre cross-sectional analyses to understand the diagnostic utility of whole exome sequencing (WES) (7, 8). HIDDEN will extend knowledge regarding the diagnostic utility and benefit of genomic testing by prospectively applying WGS in a cohort of KF in a real-world clinical setting. Understanding diagnostic yield when the strict variant analysis protocols utilised by hospital diagnostic laboratories are applied will allow seamless translation of the study results into the clinic.

Although individual inherited kidney diseases are rare, together they account for approximately 10% of KF and 70% of paediatric kidney disease (14, 15). To date, more than 500 genes have been implicated in causing various forms of kidney disease (16), with a monogenic cause being identified in around 20% of those with early-onset CKD (14) and 39% amongst those with a suspected monogenic kidney condition (12). Previous cohort studies retrospectively applied WES to early-onset (<50-year-old) and paediatric cohorts of KF patients with a diagnostic yield of between 20 and 50% (8, 17). In addition, WES and specific kidney gene panel retrospective application to adult and paediatric KF patients on transplant waiting lists similarly revealed a 10–50% diagnostic yield (7, 18, 19). The application of WES to KF cohorts of unknown genetic disease aetiology harbours some limitations, including difficulty in diagnosis due to broad phenotypic and genetic heterogeneity, and higher rate of detecting variants of uncertain significance (9). Recent cohort studies in the adult setting applying WES to 114 (20) and 92 patients (21) achieved a 56 and 59% diagnostic yield, respectively (Supplementary Material: S6 File). However, limitations of WES due to inability to detect complex deletion-insertion, copy-number variants, or variants that reside within a promotor or other intronic region cannot be detected, which may explain cases without a molecular diagnosis in these CKD cohorts. The application of WGS in the HIDDEN study has the capacity to overcome some of the limitations of WES, thus increasing diagnostic power (3).

The key novel features of this study are the prospective recruitment and application of WGS to KF patients <50 years with unknown disease aetiology in a national Australian cohort. We are hopeful that WGS application will yield a definitive diagnosis for a proportion of our cohort. It was previously estimated that the diagnostic rate may be somewhere in the realm of 5–40%, with more recent evidence demonstrating diagnostic rates of 11–51% (7, 8). The potential diagnostic value can be exemplified in the case of patients with an established histological diagnosis of Focal Segmental Glomerulosclerosis (FSGS) due to various aetiologies. FSGS accounts for 4–5% of incident KF (6), and a study by Gast et al. (22) demonstrated that an unappreciated monogenic cause for FSGS was present in 22 and 10% of cases with and without a family history of kidney disease.

It has been reported that 10–29% of patients with KF have a family history of kidney disease, with these relatives being 2–3 times more likely to have incident KF (23). These family members may be unaware of their increased risk for CKD and KF, which can arise from a combination of genetic and environmental factors. For HIDDEN patients where a molecular genetic diagnosis is made, cascade testing can be offered to family members, and may result in; early intervention or close monitoring for those already showing signs of disease or prior to symptoms/disease onset, and may have an impact on family planning (3). Whilst not clearly of direct benefit to a patient cohort already experiencing established KF, such as the cohort in this study, these potential benefits may be realised by at-risk relatives or by their treating clinicians for other patients at earlier stages of CKD including avoidance of unnecessary and burdensome investigations, in particular kidney biopsies, which carry morbidity and mortality risk (3). Testing family members allows identification of suitable live-related kidney donors for the proband. HIDDEN patients who do not receive a molecular genetic diagnosis, but have a strong family history of kidney disease, may be considered for subsequent collaborative research and/or functional genomic analysis.

A specific genetic diagnosis may lead to direct surveillance for extra-renal disease manifestations such as sensorineural hearing loss and visual impairment in Alport Syndrome, establishing social and educational supports in those conditions that significantly impact sight, vision, learning, and behaviour (3). Affected individuals of child-bearing age can also use this information to access accurate genetic counselling and reproductive genetic services prior to starting a family (3). The above benefits and utility of clinical genomic testing were demonstrated in an Australian prospective cohort study (12). Thirty-nine percent of kidney disease patients had a change in their clinical diagnosis, with 56% having a change to their clinical management, such as: 13% avoiding the need for diagnostic kidney biopsy, 44% changing surveillance, and 20% changing the treatment plan. Cascade testing was offered to 50% of families and 79% had an impact on the management of family members (12).

The potential pitfalls or unintended effects may include the following: (1) participant decisional regret to pursue genetic testing, which will be assessed by the baseline and follow up survey, (2) identifying a diagnosis that ends the diagnostic odyssey but does not have specific management strategy, (3) a genetic diagnosis may be of relevance to at-risk family members. Pre- and post-test genetic counselling is part of the enrolling clinics where support and information is provided to assist with family communication strategies, and (4) the study may not identify an answer. However, that is the current clinical *status quo* without other investigative courses of action. Pre-test genetic counselling provides information on this outcome pre-test.

Identifying an underlying genetic cause for kidney disease is the first step in identifying the group of patients and families who may benefit from interventions to reduce the burden of disease. The HIDDEN study hopes to identify a number of patients with a monogenic cause for their kidney disease that are currently undiagnosed by standard care pathways. Diagnosis positively benefits the management of their kidney disease and the management of their family. In addition, we hope to establish the diagnostic utility for this investigation pathway and demonstrate how it can be integrated into standard clinical practice.

## Author Contributions

AnM, AmM, CQ, CS, CP, and ZS conceived the study and lead it. JS, SJ, LW, RO’S, and AW assisted with coordination and operation of the study. JS, SJ, RO’S, and AnM drafted the manuscript. All coauthors reviewed and edited the manuscript. All authors contributed to the article and approved the submitted version.

## Conflict of Interest

The authors declare that the research was conducted in the absence of any commercial or financial relationships that could be construed as a potential conflict of interest.

## Publisher’s Note

All claims expressed in this article are solely those of the authors and do not necessarily represent those of their affiliated organizations, or those of the publisher, the editors and the reviewers. Any product that may be evaluated in this article, or claim that may be made by its manufacturer, is not guaranteed or endorsed by the publisher.

## Footnotes

1
https://panelapp.agha.umccr.org/panels/275/
