# Supplementary material for: The HIDDEN Protocol: An Australian Prospective Cohort Study to Determine the Utility of Whole Genome Sequencing in Kidney Failure of Unknown Aetiology
Source: Front Med (Lausanne). 2022 May 26;9:891223. doi: 10.3389/fmed.2022.891223 (PMC9204488; doi:10.3389/fmed.2022.891223)
Supplement: Supplementary file 1 [file Data_Sheet_1.pdf]

Supplementary (S) Figure 1 HIDDEN Recruitment Pathway

PROJECT HIDDEN

Identification of suitable patient for HIDDEN

1. from local patient databases and registries
2. From own clinics
3. If necessary, referral to local Renal Genetics Clinic

Nominate patient

1. Discuss patient with KidGen State clinic lead/HIDDEN review panel
2. Fills in HIDDEN recruitment proposal form - HIDDEN_NewPatient.pdf
3. Sends proposal to HIDDEN review panel OR State Clinic lead who forwards it on to review panel

Hidden recruitment proposal form

1. Contact details
2. Age, gender, ethnicity, consanguinity, Family History
3. Renal impairment – proteinuria, haematuria, renal tract imaging, biopsy, age eGRF<=15
4. Past medications
5. Past medical conditions
6. Principal phenotype inc major extra renal features
7. Mode of RRT
8. Results of any genetic testing.
9. Last referral letter

Nomination Review

HIDDEN Review Panel - A Mallett, C Quinlan, C Patel, Z Stark, A Mallawaarachchi

1. At least 2 members review all recruitment applications
2. Decide on funding source for WGS – local or AGHA HIDDEN
3. Report decision back to proposer/State Clinic lead
4. If approved, asks Regional KidGen Clinic to commence recruitment process.

Initial patient contact

1. KidGen local clinician contacts patient via phone/letter,
2. Describes study and sends link to HIDDEN information video
3. Sets up face to face consent appointment (need not be at KidGen Renal Genetics Clinic)
4. (RCH, RBWH and RAH only) Prior to appointment log into dynamic consent randomisation share drive to ascertain whether patient has been randomised to the CTRL trial

At consent appointment

1. At consent appointment study, pharmacogenomics and genomics testing and research project discussed. Clinical, Non clinical data and family history for required for REDCap and not collected on the new patient form collected
2. Consent form(s) including MBS/PBS consent and Genome.One privacy document given for either signing on the spot or to be mailed back.
3. (RCH, RBWH and RAH only) if patient allocated to CTRL dynamic consent trial then this study explained and give out login details for it. NOTE These patients will still need to fill out paper based consent form as well

After consent signed

1. Clinician to give/send patient path slip for blood collection for genome sequencing and analysis. NOTE Collection needs to be done at same hospital as recruitment
2. Clinician fills in AGHA REDCap database with data from proposal form, collected from intake and consent contacts and any clinical records requested for patient
3. Fills out Genome.One order form, ensure states HIDDEN project and who should be invoiced. Clinical WGS using target gene list KidneyOme (version #), order pharmacogenomics analysis. Attach compact PDF of download from REDCap HIDDEN instrument and any reports – e.g. biopsy, ultrasound, MRI., prior genetic testing
4. Send out HIDDEN Baseline Survey

S1 File HIIDEN Nomination Form

**Site**	Choose an item.
**Referring nephrologist**	Click here to enter text.
**Clinical Geneticist (if one involved)**	Click here to enter text.
**Preferred contact number/email**	Click here to enter text.
**Name:**	Click here to enter text.
**DOB**	Click here to enter a date.
**Sex**	Click here to enter text.
**Hospital UR No**	Click here to enter text.
**Patient Ethnicity**	Click here to enter text.
**Parental Consanguinity**	Choose an item. Click here to enter text.
**Affected family members**	Choose an item.

If yes, list affected first and second degree relatives and features

Click here to enter text.

Mandatory Investigations

**Renal tract imaging**	**attach report**
**Urinary protein**	**attach report**
**Haematuria/Urinary sediment**	**attach report**
**Age at which eGRF<= 15**	Click here to enter text.
**Any previous genetic testing**	Choose an item.
	**If yes attach report**
**If renal biopsy has been done:**	**attach report**
**Past medical conditions**	**attach report or enter below**

Click here to enter text.

Past medications **attach report or enter below**

Click here to enter text.

Principal phenotypic features/relevant investigations, e.g. imaging/biopsy findings:

Aim for 5-10 positive/negative (e.g. cysts, interstitial fibrosis, haematuria, proteinuria, abnormal glomerular basement membrane).

Include major extra renal features.

Click here to enter text.

Attach last clinic letter.

Mode of RRT Choose an item.

***All cases will have KidneyOme analysis* and pharmacogenomics**

S2 File Participant Information Statement/Consent Form HIDDEN Renal Genetics

[Insert site name]

**Title**	*Australian Genomics Health Alliance: Preparing Australia for Genomic Medicine*
**HREC Number**	*2016.224*
**Coordinating Principal Investigator**	*Prof Kathryn North, Program Lead, Australian Genomics*
**Site Principal Investigator**	*[Name]*
**Version no:** *1*	**Version date:** *10 January 2019*

This information statement describes the research project. It is divided into four sections:

Section 1 – Information about the research project

Section 2 – Understanding genomic testing and pharmacogenomic testing

Section 3 – Understanding your experiences and measuring the impact of your genomic test

Section 4 – Further research

Questions to ask your doctor/genetic counsellor

Once you have read this form, you may wish to ask your doctor/genetic counsellor these questions:

- What is the chance that the genomic test will identify the cause of my/my child's condition?
- How long will it take to get a result?
- Who will give me the result and how?
- Where will my genomic test be performed?
- What is the cost to me (if any) of my genomic test?
- What (if any) are the implications for other members of my family if I have this test?
- What is the chance of this test finding something that is unrelated to my/my child's current health condition?

Section 1 – Information about the research project

a) Introduction

We would like to invite you to participate in a research project that is explained below. This form explains the steps and procedures involved in the project. Knowing what is involved will help you decide if you want to take part. Please read this Information Statement carefully.

Participation in this research is voluntary. You will receive the best possible care whether or not you take part. Before you decide if you want to take part or not, you can ask us any questions you have about the project. You may also want to talk about the project with your family, friends or health care provider.

If you decide you want to take part in the research project, you will be asked to sign the consent form. By signing it you are telling us that you:

- Understand what you have read.
- Consent to take part in the research project.
- Consent to the use of your personal and health information as described.
- Have had a chance to ask questions and received satisfactory answers.

You will be given a copy of this Participant Information Statement and the Consent Form to keep.

b) About the Australian Genomics Research Project

This research project is looking at how useful genomic testing is for the diagnosis and/or management of a number of medical conditions and to determine how it affects patient care. This will help to find out when we should use this test and the most appropriate and cost-effective way of providing genomic testing to patients in the future.

c) Why am I being asked to participate in this research project?

You have been asked to take part in the study because you have one of the conditions for which we are trying to work out if genomic testing is useful.

d) What does participation in the research involve?

Visit 1 – Consent

- You will be asked if you are interested in participating in the research project.
- The study purpose and procedures are explained on the day of your visit. The consent form may be signed during this visit or you may discuss the study and provide your consent with a genetic counsellor over the phone or at another appointment.
- Collection of your blood sample at this visit, or at a later date if it is more convenient. This sample will be used to carry out your genomic test.

Survey 1: Pre-Test

You may be sent an email requesting you to do a survey, which you will complete online.

Visit 2 –Return of results

Genomic testing may or may not find a diagnosis. If a diagnosis for your condition is determined you will be contacted by a member of the KidGen Renal Genetics Team to make an appointment to discuss it. Following this appointment, your results will be sent to your treating clinician along with any significant results from the pharmacogenomics analysis which your treating clinician may use to tailor the medications prescribed for you to your genetic makeup. In some instances, we may send your samples or genomic data on to continued research to assist with research into your condition. (Section 4).

Contact if no conclusive result

If there is no, or an inconclusive, result your samples or genomic data may go on to continued research to try to find a diagnosis and/or understand your condition (Section 4). At this stage we may contact you to ask if family members with renal disorders might be interested in providing blood samples for the study to further inform the research.

If after 12 months we are still unable to find a diagnosis for your condition, you and your treating clinician will be contacted and advised of this outcome. Any significant findings from the pharmacogenomics analysis will be returned to your treating clinician who may use it to tailor the medications prescribed for you to your genetic makeup.

Survey 2: Post-Test (Over a month after return of results)

You may be sent an email requesting you to do a follow-up survey about your experience with genomic testing. Again, you will complete it online.

There are no out-of-pocket costs associated with participating in this research project including the initial genomic test. Additional testing which may be suggested by your doctor may not be included as part of this research project. There may be a cost to the family for these tests. You will not be paid for your participation.

e) Do I have to take part in this research project?

Participation in this research project is voluntary. Please talk to your clinician about alternative testing options if you decide not to participate.

You are free to withdraw from the project at any stage. If you withdraw before testing and data is collected, we will not continue. If you withdraw after testing and data is collected we will use any information already collected unless you tell us not to. However, if your data has already been shared it may not be possible to retrieve or remove all your data.

To opt out, please contact the clinical contact person listed in section 1(k) below.

f) How will the results be provided to me?

If a diagnosis for your condition is determined through testing, you will be contacted by a member of the KidGen renal genetics team to make an appointment to discuss this result. Following this appointment, the renal genetics team will send a letter to your treating clinician to advise them of the result. Testing may not result in a diagnosis. If after 12 months we are still unable to find a diagnosis for your condition, you and your treating clinician will be contacted to advise of this outcome. Research results beyond the diagnostic report may be provided if available. This could include a summary of the impact of the genomic testing for the conditions that are being studied as part of this research.

g) What will happen to information about me?

Your participation in this research project and any information obtained in connection with this research project that can identify you ("**identified**" information) will remain confidential, except as required by law. All study data will be stored in secure, controlled-access databases that meet international security standards and Australian laboratory accreditation requirements. Only the study doctors, study researchers and personnel working directly with this study will have access to the database. However, if you choose, information about your participation in this research project may also be recorded in your health records.

Providing consent for genomic testing also allows for the sharing of your sample, genomic data and related health information to advance scientific knowledge. Your information will be shared in a way that protects your privacy ("**anonymised**"). This may include sharing on large databases to help improve understanding of related conditions by comparing your results to those from other people.

Your "**de-identified**" information and sample will only be shared when safeguards are in place to help protect your privacy. Personal identifiers will be removed (including your name, date of birth, and address) and stringent security measures will help prevent unauthorised access or misuse. Sharing will also involve the minimum, necessary information. These safeguards make it difficult to know whether the information is about you or other people; however, there is always a very small chance that you might be re-identified. Given that the potential to identify you is significantly reduced, you are unlikely to directly benefit from this sharing.

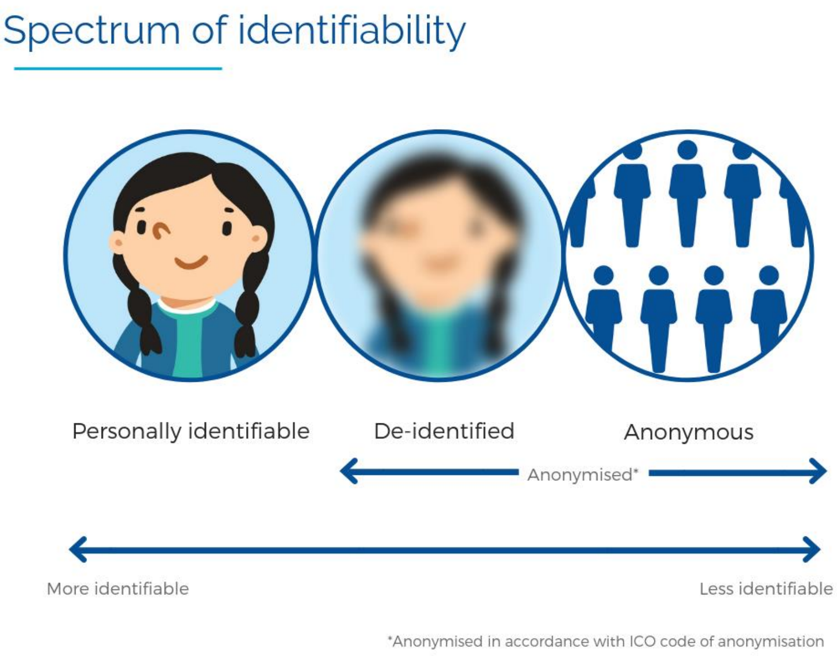

Your health records and any information collected and stored by the study doctors during the research project may be reviewed for the purpose of verifying the procedures and the study data. This is to ensure that the study team are complying with the approved study design and procedures. This review may be done by the ethics committee that approved this research project, regulatory authorities, or as required by law. In these circumstances, these parties will review only, not collect or record, your personal information. By signing the consent form, you authorise the release of, or access to, this confidential information.

In accordance with relevant Australian and/or State privacy and other relevant laws, you have the right to request access to your information collected and stored by the study team. You also have the right to request the correction of any information with which you disagree. Please contact the study team member named at the end of this document if you would like to request access to your information.

It is anticipated that the results of this research project will be published and/or presented in scientific and medical meetings. In any publication and/or presentation, information will be provided in such a way that you cannot be identified. Data will be stored for at least 5 years after the publication of the results to comply with the recommendations of the Australian Code for Responsible Conduct of Research.

h) What will happen to the samples collected for these studies?

Your sample will be sent directly to the laboratory for DNA extraction and testing. In the diagnostic setting, your samples will be identifiable as a requirement of sample tracking. Your samples will be kept indefinitely unless you request their destruction.

We would like to store your health information, research data and DNA sample for use in any future research studies that may or may not be related to the original research project. Further information can be found in Section 4.

i) Who is funding this research project?

This research is being led by Australian Genomics, a collaboration of major research institutions, universities and clinical services across Australia. It is funded by the National Health and Medical Research Council. The study doctors and researchers are employees of the major collaborating institutions, universities and clinical services.

j) Who has reviewed the research project?

The ethical aspects of this research project have been reviewed by the Human Research Ethics Committee of Melbourne Health. This project will be carried out according to the *National Statement on Ethical Conduct in Human Research (2007)*.

k) Further information and who to contact

If you want any further information concerning this project, you can contact:

Clinical contact person

Name	*[Name]*
Position	*[Position]*
Telephone	*[Phone number]*
Email	*[Email address]*

If you have any complaints about any aspect of the project, the way it is being conducted or any questions about being a research participant in general, then you may contact:

Reviewing HREC approving this research and HREC Executive Officer details

Reviewing HREC name	*Melbourne Health*
HREC Executive Officer	*HREC manager*
Telephone	*03 9342 8530*
Email	*research@mh.org.au*

Local HREC Office contact (Single Site - Research Governance Officer)

Name	*[Name]*
Position	*[Position]*
Telephone	*[Phone number]*
Email	*[Email address]*

Section 2 – Understanding Genomic Testing and Pharmacogenomic Testing

a) About the test – What is Genomic testing?

Our bodies are made up of billions of cells. In most of our cells, we have a complete copy of our genetic information (genome). We all have about 20,000 genes in our genome. Our genes are made of DNA and contain the instructions for growth and development of the body. Until recently, doctors and scientists were only able to test one gene at a time. New technology allows us to test all of our genes at once (genomic testing). Each person's genome contains many genetic differences (variants). Most of these are harmless and do not change how the gene works in the body. Genomic testing is done to find gene variants that do change how a gene works and cause health or developmental conditions.

b) Potential outcomes of genomic testing

Your doctor or genetic counsellor will discuss the outcomes of genomic testing including:

- Finding a variant that is the cause of the condition.

- Finding a variant of unknown significance (VUS). The effect of a VUS is unknown. Sometimes testing in other family members for the VUS may help to understand if it could be the cause of a condition. The understanding of the VUS may change over time.
- No gene variants found that could explain a genetic condition. Reasons for this include:
    - the variant causing the condition cannot be found by the test;
    - the gene causing the condition was not tested;
    - the gene causing the condition is not yet known;
    - the condition may not have a genetic cause.

  Future testing may help clarify this, but the timing for this is unknown.

c) Potential benefits of genomic testing

Some people wish to have genomic testing to find a genetic diagnosis to help them understand their or their child's condition. A genetic diagnosis can also sometimes help families to access support and services that they need, and to plan for the future. A genetic diagnosis may also help health professionals manage a condition.

A genetic diagnosis may provide families with information about the chance of having another child with the same condition. Sometimes, the genomic test result in one person may also be important for the care of their relatives.

Genomic testing can lead to a diagnosis in 30-50% of people with rare genetic conditions. If a diagnosis doesn't happen today, the genomic test result could be looked at in the future as our understanding improves.

It is important to remember that genomic testing is not a general health test and will not identify all gene changes that could contribute to health problems that may develop in the future.

d) Potential risks of genomic testing

Incidental findings

In genomic testing, we are looking at many genes all at once and so there is a small chance they might see a variant in a gene that is not related to your health condition. This is called an incidental finding. It is a variant in a gene not related to the reason for doing the genomic test, but could be important to know about for your health. If your doctor decides that these incidental findings have important consequences for you or other family members, they will raise it with you. If medical follow up is required as a result of an incidental finding, your doctor or genetic counsellor will assist you by making appropriate referrals, if necessary. Your doctor or genetic counsellor will be able to give you some examples of incidental findings.

Insurance

In Australia, genomic testing will not alter your ability to get health insurance or your health insurance premiums. Genomic testing in you or your child could affect how easy it is for you or other family members to get income protection, travel or life insurance, or the price of your premium. An existing diagnosis may already affect your ability to obtain these kinds of insurance. Industry regulation prevents insurers from asking relatives for your genetic test results, and you cannot be requested to have a test by an insurer. Your healthcare provider will not provide your results to an insurance provider without your permission.

Further details can be found at: http://www.genetics.edu.au/Publications-and-Resources/Genetics-Fact-Sheets/FactSheet23A.

Blood sample collection

There are no major risks associated with a blood sample collection. It is possible you may feel some discomfort during the blood test. It is possible there may be some bruising, swelling or bleeding where the needle enters the skin. Some people can feel a little light-headed when blood is taken.

e) Sharing results to help family members

Genetic services will not usually contact your relatives. However, your relatives may be referred to genetic services to arrange testing for themselves or their children when you tell them, or, when they find out there is a genetic condition in the family. With your permission, your test results may be released to another genetics service to help with the care of other family members. As genetic changes are unique in families, it is helpful for genetic services to be able to share information (for example – exactly what genetic change is present in a family), so that the correct testing can be offered to others in the family who may be at risk. All efforts will be made to ensure that your identity is not revealed to those family members unless you wish to provide that information.

f) Pharmacogenomic testing

Irrespective of whether a diagnosis is obtained through the genomic testing, pharmacogenomic testing will be undertaken on your DNA sample and any findings will be included in the clinical report. Pharmacogenomics is used to analyse your DNA sample to look for genes that are associated with how medications are absorbed, metabolised and excreted. This information may be able to be used by your treating clinician to tailor the medications prescribed for you to your genetic makeup.

Section 3 – Understanding your experiences and measuring the impact of your genomic test.

One of our aims is to understand the costs and benefits of genomic testing for patients and their families, the government, society and the health system itself. To do this we need to collect information from you and about you.

a) How will we collect this information?

Survey Participation

As part of this study, we would like you to complete questionnaires on the health, financial, social and emotional impacts of living with your condition, your views on genomic testing and experience related to this testing. Examples of the types of information asked may be about the impact of your condition on your ability to work, your quality of life, your family planning decisions and your relationships. To find out if genomic testing has changed anything for you, you will be asked to complete questionnaires before and after your testing.

Survey 1: Pre-test: you will be asked to complete this survey around the time the genomic testing is ordered.
Survey 2: Post-test: you will be asked to complete this survey around one month after receiving the genomic test result.

Access to your health data

As part of your consent, we will gather information about your health condition and health care (such hospital visits, medicines prescribed, etc). Researchers may also ask you directly for more details. This will allow us to access healthcare information about you: your condition, tests you have had, treatments you have received, any other health problems and history of related health problems in your family. This helps us to learn whether genomic testing is useful for you, or to those living with your condition.

Hospital and Emergency Datasets

This study will request access to your hospital and emergency records that are related to your condition. The collection of this information is usually required by law and is securely stored by the service or agency that collects it. In brief, we will supply the data linkage agency with some of our identifying information such as your name, date of birth, and address. The data linkage agency will then create a unique ID for you and send it to hospital and emergency data custodian. Your hospital and emergency records will be merged with the unique ID and be provided to us.

We will access information from 2007 (or the date of your birth if later) through to the end of the study, which is up to 14 years. This will provide us with data about healthcare resources you have used, to help us to study the potential economic impact of genomic testing. We will collect information about you from the following databases through third party data linkage agencies:

**Hospital Datasets**	**Emergency Department Datasets**
- VIC Admitted Episodes Dataset<br>- NSW Admitted Patient Data Collection<br>- ACT Admitted Patient Care<br>- QLD Hospital Admitted Patient Data Collection<br>- WA Hospital Morbidity Data System<br>- SA Inpatient Hospital Separations<br>- NT Inpatient Activity<br>- TAS Public Hospital Admitted Patient Collection	- VIC Emergency Minimum Dataset<br>- ACT/NSW/SA/WA Emergency Department Data Collection<br>- QLD Emergency Department Information System<br>- NT Emergency Department Activity Collection<br>- TAS Public Hospital Emergency Department Presentations

Medicare Benefits Schedule (MBS) and Pharmaceutical Benefits Scheme (PBS) data

You will be asked to fill out a separate consent form that authorises the research investigators to access to your MBS and PBS data. Medicare collects information on your medical visits and procedures, and the associated costs, while the PBS collects information on the prescription medications you have filled at pharmacies. The consent form is sent securely to the Department of Human Services who holds this information confidentially. This information will only be used by the current study and will not be shared.

Other opportunities

We may offer you the opportunity to participate in focus groups or workshops so that we can ask you questions about your experience with genomic testing.

b) What are the possible benefits of sharing your information?

There may be no direct benefit to you from taking part in this research. The outcomes may provide valuable information about whether this test should be part of routine care for patients with your condition in the future.

c) What are the possible risks and disadvantages of sharing your information?

Being asked about your experience of testing, or the impact of a genetic condition on your emotional, social and financial situation may be upsetting for some people, but your genetic health care provider or specialist is available to discuss any concerns with you.

d) What will happen to information about me?

Survey data will not be labelled with any personal details (such as name, date of birth or hospital number), but instead will be identified by a unique study identifier number (UIN). Only the study

doctors and their relevant research staff will be able to re-identify the UIN and return information back to you.

Data related to MBS, PBS, and hospital and emergency datasets will be stored on secured servers (located at the Murdoch Children's Research Institute) and access to the data will be limited to authorised researchers in this study via assigned login password. All data related to MBS, PBS, and hospital and emergency datasets will be destroyed after 7 years from the publication of the final project report. Hard copies will be shredded and destroyed by a secured destruction service provider. Non-identifiable data will be stored indefinitely as the economic model will be critically appraised by the scientific community. Updating and running of the model will require access to the non-identifiable data files.

Section 4 - Further Research

We may use your samples and data in extended research as part of this study without further consent. This may occur:

- when no cause is found as part of the initial testing and/or
- further investigation is required to understand your condition.

All researchers involved are required to follow the relevant laws (including privacy and security requirements) and ethical guidelines for biomedical and health research.

a) What data and samples will be shared and will results be returned?

Your de-identified data and samples will be shared however if need, they can be re-identified. This will be important if there are findings from this research that have implications for your future clinical care, it may be possible to contact you, so that your result can be returned. However, this research does not guarantee direct benefits to you.

b) Additional Samples

In some cases, we may be able to use the samples you have provided for the initial genomic test, however, we may also need to access additional samples that were collected as part of your standard clinical investigations (including blood cells, skin cells, hair follicles and/or tissue related to your condition). If there are available samples we will contact the laboratory storing them directly. We will provide the laboratory with your consent to this study to access them and you will not be required to fill out another consent form. We may collect excess tissues if you are having surgery for your condition to look for possible genetic causes that may only be found in those tissues.

c) What will these samples be used for?

This research may include more in-depth genomic sequencing and/or other laboratory studies on your samples, either in Australia or overseas, to see if a cause can be found for your condition.

Advances in technology mean we can use new tests to give us more information to better understand the structure of genes and how they work.
Examples of what we may use your samples for:

- perform functional studies: this research seeks to understand the contribution/function of a single gene and impact of a possible genetic variant.
- generate an induced pluripotent stem cell (iPSCs) line: these are usually generated from skin cells. iPSCs have similar properties to embryonic stem cells. They can self-renew and form all cell types in the body. We can use them to make the specific type of cell that is not working properly in the affected individual.

d) Optional Additional Research

Other doctors and scientists at this and other medical and research centres may use your de-identified health information, research data and/or sample in further research with the goal to improve health outcomes and develop new treatments. Results from research carried out by other approved researchers will not be returned to you as they will not have access to your identifying information.

Consent Options

1. You will be asked if you agree to share your data (health-related, genomic and self-reported information) and sample/s for use in ethically approved research outside this study. If you do agree to this you will not be contacted for further consent, and your data and sample/s will be shared in a **de-identified** way
2. We may like to contact you in the future about the possibility of participating in other ethically approved research projects. This is optional and you can indicate your willingness to be involved in such research on the consent form.

- Researchers may publish the results of their research in medical journals. They may also present their results at scientific meetings. It is important for scientists and doctors to share results to help research advance as quickly as possible. You will not be identified in any of this.
- We will not allow access to any data for marketing or insurance purposes.
- There may be no direct benefit of the option of additional research. Other people might benefit if researchers learn more by using your health information, research data and/or samples.

The CTRL website – a digital consent and engagement tool:

We may ask you to use a website called CTRL to manage your choices about being in the study and to allow you to engage with the research in an ongoing basis.

If you are offered and agree to be part of the trial of the CTRL website, you will be shown how to register by the person that takes you through this consent form. The aim of this website is to give you more choices about your consent to be in the study and ongoing contact with the study. For example, you will be offered more choices about which types of additional research projects we share your genomic and other health information with. We will also be able to provide you with research updates. This could lead to a better experience in the research project for you. We will find out whether using this website improves your experience of being involved in the research by recording how you use the website and through your answers to specific questions in the participant surveys you will complete. This study has been approved by the Royal Children's Hospital HREC (study number HREC/18/RCHM/191).

Consent Form for Genomic Testing, Pharmacogenomic Testing and Participating in Research

It is my choice to have genomic testing, pharmacogenomics testing and participate in the Australian Genomics research project. I can say yes or no to the options on this form.

I, ____(patient and/or parent/guardian names),

understand that my DNA will be tested by panel/exome/genome to look for changes in genes that may

be associated with ____(condition). My

DNA will also be tested to look for genetic factors that may influence how my body processes

medications (pharmacogenomics testing). I have been asked to participate in the Australian Genomics

HIDDEN Renal Genetics Flagship.

About the Test

- Genomic testing is done on DNA from my blood, saliva or tissue.
- Genomic test results are based on current knowledge, which may change in the future.
- Pharmacogenomic testing results are based on current knowledge, which may change in the future.

Potential Outcomes

I understand that:

- This test may find a cause for the condition(s).
- This test may <u>not</u> find a cause for the condition(s).
- The result may be of *'unknown significance*', which means it cannot be understood today.
- There is a chance that genomic testing could find other medical conditions (incidental findings).
- This test will not predict all future health problems.
- Genomic testing may identify unexpected family relationships.
- Further testing or information sharing may be needed to finalise the result.
- Pharmacogenomic testing may or may not find genetic factors that influence how your body processes medications.

Results

I understand that:

- I will be told the results by a doctor or a genetic counsellor who will arrange appropriate follow up care, as necessary.
- Results may have implications for the health/genetic risks of my family members.
- Results from these tests may affect my ability to obtain some types of insurance.
- The results will be available to health professionals involved in my care.

- Results are confidential and will not be released beyond the study team without my consent, unless allowed by law.
- Results of this testing may be used for the healthcare of my family members.
- In the event of my death, my results can be released to:

**Name**	**Contact**

Laboratory Sample and Data Storage

I understand that:

- Sometimes a second DNA sample is needed.
- The **sample, genomic results and test report** will be handled according to applicable law and standard laboratory practice.
- I request my sample(s) be destroyed after the required storage time:

  □ **Yes** □ **No**

Data and Sample Sharing

I understand that:

- The **identified** sample, genomic results, test report and health related data will only be used by those involved in my care and Australian Genomics study members, unless required or allowed by law.
- The **de-identified** sample, genomic results and related health information is labelled only with my coded study number. It may be shared and stored with other researchers when no cause is found from the initial test and/or to further understanding of my condition. This research may be linked back to me if relevant to my care.
- **Anonymised** genomic data and associated health information may be shared for the purpose of advancing knowledge and I am unlikely to gain any personal benefit from this.

Participating in Research

I understand:

- The purposes, procedures and risks of the research project.
- I am free to withdraw at any time during the project without any impact on my future health care.
- Health related data may be collected for up to 10 years following genomic testing and that information will be used only by Australian Genomics, and not shared further.
- Further research may be initiated to understand my genomic results and/or condition. Additional samples may be requested.
- I may be asked to complete survey(s) regarding my understanding and experience of genomic testing, costs associated with living with my condition.

I provide consent for:

- My doctors, other health professionals, hospitals, federal and state agencies to release my re-identifiable information to Australian Genomics for the purposes of this project.
- The laboratory undertaking the testing to release my identifiable genetic and genomic data associated with this study to Australian Genomics. I acknowledge that Australian Genomics will then have responsibility for the appropriate use and storage of the released data.
- My samples and genomic data may be used in extended research studies when no cause is found from the initial test and/or to further understanding of my condition.

Optional Consent for Future Research

I also provide consent for:

- Sharing the sample, genomic results and related health information with third parties other than Australian Genomics, for ethically approved research, into the same or a related condition, where it is possible for me to be re-identified. This allows information to be returned to me where appropriate, but participation may not have direct benefits to me or my family.

  □ **Yes** □ **No**
- Being contacted about other kinds of genomic research in the future. If interested in taking part, I would be asked to sign a separate consent form.

  □ **Yes** □ **No**

I have had enough time to consider the information in this consent form and have:

- Had genomic testing and pharmacogenomics testing explained to me by a health professional.
- Been given written information about genomic testing, pharmacogenomics testingand this research study.
- Been able to ask questions until I am satisfied with the answers.
- Been offered a copy of this consent form.

I provide consent to have genomic testing, pharmacogenomic testing and participate in research as summarised in this form.

□ **Do not send reports to My Health Record**

Participant

Signature: ___________________________________ Date of Signature:__________________

Print Name: __________________________________ Date of Birth:_______________________

Address:____

Email:____

Optional Witness – confirming verbal consent

Signature: ___________________________________ Date of Signature:__________________

Print Name: __________________________________

Health Professional

I have explained the project to the participant who has signed above, and believe that they understand the purpose, extent and possible risks of their involvement in this project.

Signature: ___________________________________ Date of Signature:__________________

Print Name: ________________________________

Note: All parties signing the Consent Form must date their own signature.

NOTE: Copy of this consent is to be sent to testing laboratory with sample.

Affix identifier information here

S3 Hidden REDCap Phenotype data collection

Study Number: ______________________

Serology features. Select all that apply.
only select those features that pre-date renal failure - if known

- ☐ NONE
- ☐ UNKNOWN (add details below)
- ☐ Yes but not in this list (add details below)
- ☐ Nephrolithiasis
- ☐ Calcium oxalate nephrolithiasis
- ☐ Renal salt wasting
- ☐ Renal sodium wasting
- ☐ Renal potassium wasting
- ☐ Renal magnesium wasting
- ☐ Impaired renal uric acid clearance
- ☐ Renal chloride wasting
- ☐ Renal calcium wasting
- ☐ Renal phosphate wasting
- ☐ Renal hypophosphatemia
- ☐ Increased renal tubular phosphate reabsorption
- ☐ Decreased renal tubular phosphate excretion
- ☐ Parathormone-independent increased renal tubular calcium reabsorption
- ☐ Low alkaline phosphatase of renal origin

(HPO term - select 'Yes but not in this list' if not in the lists)

For the definition of each of the Serology HPO terms see here

[Attachment: "Serology HPO definitions.pdf"]

Please add details if unknown ______________________

List any other serological or biochemical findings not listed above
DO NOT copy and paste reports here. These should be scanned and uploaded in the Upload document instrument ______________________

Urinalysis findings. Select all that apply
only select those features that pre-date renal failure - if known

- ☐ NONE
- ☐ UNKNOWN (add details below)
- ☐ Yes but not in this list (add details below)
- ☐ No Proteinuria
- ☐ Proteinuria
- ☐ Mild proteinuria
- ☐ Moderate proteinuria
- ☐ Nephrotic range proteinuria
- ☐ Low-molecular-weight proteinuria
- ☐ No hematuria
- ☐ Hematuria
- ☐ Macroscopic hematuria
- ☐ Microscopic hematuria
- ☐ Abnormality of the renal tubule
- ☐ Renal tubular dysfunction/Renal tubular defect
- ☐ Proximal tubulopathy/Proximal renal tubule defect
- ☐ Global proximal tubulopathy
- ☐ Non-acidotic proximal tubulopathy
- ☐ Proximal renal tubular acidosis
- ☐ Bicarbonate-wasting renal tubular acidosis
- ☐ Generalized distal tubular acidosis
- ☐ Distal renal tubular acidosis
- ☐ Renal tubular acidosis
- ☐ Renal Fanconi syndrome
- ☐ Renal aminoaciduria
- ☐ Reduced ratio of renal calcium clearance to creatinine clearance
- ☐ Parathormone-independent increased renal tubular calcium reabsorption
- ☐ Increased renal tubular phosphate reabsorption
- ☐ Decreased renal tubular phosphate excretion
- ☐ Renal tubular lysine transport defect
- ☐ Calcium oxalate nephrolithiasis
- ☐ Abnormality of renal resorption
- ☐ Impaired renal concentrating ability
- ☐ Impaired urinary acidification

(HPO term - select 'Yes but not in this list' if not in the lists)

For the definition of each of the Urinalysis HPO terms see here

[Attachment: "Urinalysis HPO definitions.pdf"]

Please add details if unknown

____

List any other urinalysis findings not listed above.
DO NOT copy and paste reports here. These should be scanned and uploaded in the Upload document instrument

____

Imaging findings. Select all that apply
only select those features that pre-date renal failure - if known

- ☐ NONE
- ☐ UNKNOWN (add details below)
- ☐ Yes but not in this list (add details below)
- ☐ Hyperechogenic kidneys
- ☐ Absence of renal corticomedullary differentiation
- ☐ Reduced renal corticomedullary differentiation
- ☐ Abnormal renal corticomedullary differentiation
- ☐ Bilateral normal kidney length
- ☐ Bilateral renal atrophy
- ☐ Unilateral normal kidney length
- ☐ Unilateral renal atrophy
- ☐ Nephrocalcinosis
- ☐ Nephrolithiasis
- ☐ No renal cysts
- ☐ Solitary renal cyst
- ☐ Renal cyst/Cystic kidney disease
- ☐ Renal corticomedullary cysts
- ☐ Renal cortical cysts
- ☐ Multiple small medullary renal cysts
- ☐ Multiple renal cysts
- ☐ Polycystic kidney dysplasia/Enlarged polycystic kidneys
- ☐ Enlarged kidney
- ☐ Cystic renal dysplasia
- ☐ Multicystic kidney dysplasia/Multicystic dysplastic kidney
- ☐ Renal duplication/supernumerary Kidney
- ☐ Duplicated collecting system
- ☐ Horseshoe kidney
- ☐ Ectopic kidney/Displaced kidney
- ☐ Crossed fused renal ectopia/Ectopic kidney with fusion
- ☐ Pelvic kidney
- ☐ Abnormal localization of kidney
- ☐ Renal malrotation
- ☐ Bilateral renal dysplasia
- ☐ Unilateral renal dysplasia
- ☐ Unilateral renal hypoplasia
- ☐ Bilateral renal hypoplasia
- ☐ Bilateral renal agenesis
- ☐ Unilateral renal agenesis
- ☐ Duplication of renal pelvis
- ☐ Partially duplicated kidney
- ☐ Abnormality of the renal pelvis
- ☐ Dilatation of the renal pelvis
- ☐ Fetal pyelectasis/Fetal renal pelvic dilatation
- ☐ Renal angiomyolipoma
- ☐ Abnormal renal artery morphology
- ☐ Abnormality of renal cortex morphology
- ☐ Abnormality of renal calyx morphology
- ☐ Abnormal renal morphology
- ☐ Renal medullary pyramid hypoplasia
- ☐ Dilatation of renal calices
- ☐ Renal diverticulum
- ☐ Abnormality of the renal medulla

(HPO term - select 'Yes but not in this list' if not in the lists)

For the definition of each of the Imaging HPO terms see here

[Attachment: "Imaging HPO Definitions.pdf"]

Please add details if unknown

____

List any other imaging findings not listed above
DO NOT copy and paste reports here. These should be scanned and uploaded in the Upload document instrument

____

Biopsy findings. Select all that apply
only select those features that pre-date renal failure - if known

- ☐ NONE
- ☐ UNKNOWN (add details below)
- ☐ Yes but not in this list (add details below)
- ☐ Immunofluorescence not performed
- ☐ Electron Microscopy not performed
- ☐ Tubular atrophy
- ☐ Tubulointerstitial fibrosis
- ☐ Renal fibrosis
- ☐ Renotubular dysgenesis
- ☐ Renal cortical atrophy
- ☐ Renal cortical microcysts
- ☐ Multiple glomerular cysts
- ☐ Abnormality of renal glomerulus morphology
- ☐ Glomerulopathy
- ☐ Glomerulosclerosis
- ☐ Focal segmental glomerulosclerosis
- ☐ Glomerulonephritis
- ☐ Membranoproliferative glomerulonephritis
- ☐ Membranous nephropathy
- ☐ Minimal change glomerulonephritis
- ☐ Diffuse mesangial sclerosis
- ☐ Global glomerulosclerosis
- ☐ Crescentic glomerulonephritis
- ☐ Nephrocalcinosis
- ☐ Nephritis
- ☐ Thickening of the glomerular basement membrane
- ☐ Glomerular subendothelial electron-dense deposits
- ☐ Tubular basement membrane disintegration
- ☐ Thin glomerular basement membrane
- ☐ Diffuse glomerular basement membrane lamellation
- ☐ Renal juxtaglomerular cell hypertrophy/hyperplasia
- ☐ IgA deposition in the glomerulus
- ☐ Glomerular C3 deposition
- ☐ Renal amyloidosis
- ☐ Abnormal renal corpuscle morphology
- ☐ Abnormal glomerular capillary morphology
- ☐ Abnormal morphology of Bowman capsule

(HPO term - select 'Yes but not in this list' if not in the lists)

For the definition of each of the Biopsy HPO terms see here

[Attachment: "Biopsy HPO Definitions.pdf"]

Please add details if unknown

____

List any other biopsy findings not listed above.
DO NOT copy and paste reports here. These should be scanned and uploaded in the Upload document instrument

____

Other general/non-specific renal features. Select all that apply	☐ NONE<br>☐ UNKNOWN (add details below)<br>☐ Yes but not in this list (add details below)<br>☐ Abnormality of the kidney<br>☐ Nephropathy<br>☐ Abnormality of the renal collecting system<br>☐ Abnormality of renal excretion<br>☐ Abnormal renal morphology<br>☐ Abnormal renal physiology<br>☐ Renal cell carcinoma<br>☐ Acute kidney injury<br>(HPO term - select 'Yes but not in this list' if not in the lists)
For the definition of each of the Generic HPO terms see here<br><br>[Attachment: "Generic HIDDEN HPO Definitions.pdf"]	
Please add details if unknown	__________________________________
List any other renal specific findings not listed above.<br>DO NOT copy and paste reports here. These should be scanned and uploaded in the Upload document instrument	__________________________________
Are there any non renal phenotypic features ?	○ Yes ○ No
Audiology	○ Yes ○ No
Audiology features	__________________________________
Ophthalmology	○ Yes ○ No
Ophthalmology features	__________________________________
Endocrinology	○ Yes ○ No
Endocrinology features	__________________________________
Gastroenterology	○ Yes ○ No
Gastroenterology features	__________________________________
Skeletal	○ Yes ○ No

Skeletal features	__________________________________
Cardiology	◯ Yes ◯ No
Cardiology features	__________________________________
Respiratory	◯ Yes ◯ No
Respiratory features	__________________________________
Neurology	◯ Yes ◯ No
Neurology features	__________________________________
Dermatology	◯ Yes ◯ No
Dermatology features	__________________________________
Haematology	◯ Yes ◯ No
Haematology features	__________________________________
Immunology	◯ Yes ◯ No
Immunology features	__________________________________
Oncology	◯ Yes ◯ No
Oncology features	__________________________________
Reproductive	◯ Yes ◯ No
Reproductive features	__________________________________
Developmental/Learning/Behavioural issues	◯ Yes ◯ No

Developmental/Learning/Behavioural issues details	____________________
Dysmorphology	◯ Yes ◯ No
Dysmorphology features	____________________
Are there any other non renal features?	◯ Yes ◯ No
Other non-renal features	____________________
Growth parameters	◯ Yes ◯ No
Growth features	____________________
Age at which CKD first diagnosed	◯ Unknown<br>◯ < 2 years<br>◯ between 2 years and 5 years<br>◯ between 5 years and 18 years<br>◯ between 18 years and 30 years<br>◯ > 30 years
Age when first saw renal physician/nephrologist	◯ Unknown<br>◯ < 2 years<br>◯ between 2 years and 5 years<br>◯ between 5 years and 18 years<br>◯ between 18 years and 30 years<br>◯ > 30 years
Stage of CKD when first diagnosed	◯ Unknown<br>◯ Stage 1 chronic kidney disease<br>◯ Stage 2 chronic kidney disease<br>◯ Stage 3 chronic kidney disease<br>◯ Stage 4 chronic kidney disease<br>◯ Stage 5 chronic kidney disease
Age at which eGRF< 15 (CKD 5)	◯ unknown<br>◯ < 2 years<br>◯ between 2 years and 5 years<br>◯ between 5 years and 18 years<br>◯ between 18 years and 30 years<br>◯ > 30 years
Age (in months) at which eGRF< 15 (CKD 5)	____________________<br>(number of months only)
Age (in years) at which eGRF< 15 (CKD 5)	____________________<br>(whole years only)

Current RRT	☐ None, CKD5 - Pre RRT<br>☐ Haemodialysis<br>☐ Peritoneal dialysis<br>☐ Transplant (answer below)
Previous RRT	☐ None/same as current<br>☐ Haemodialysis<br>☐ Peritoneal dialysis<br>☐ Transplant
Age commenced RRT?<br>Enter whole number only. If dialysis not commenced enter NA.	______________________<br>(round to nearest whole year. If less than 1 put 0)
Transplant	◯ Yes, more than one ◯ Yes, only one<br>◯ No
Year of transplant	______________________
Source of transplant	◯ donor brain death<br>◯ donor cardiac death<br>◯ living related donor<br>◯ living non related donor
Year of current transplant	______________________
Source of current transplant	◯ donor brain death<br>◯ donor cardiac death<br>◯ living related donor<br>◯ living non related donor
Relationship to donor	______________________
Number of previous transplants, not including current transplant	◯ 1 ◯ 2 ◯ 3 ◯ 4
Year of previous transplant	______________________
Source of previous transplant	◯ donor brain death<br>◯ donor cardiac death<br>◯ living related donor<br>◯ living non related donor
Reason for failure of previous transplant	____________________________
Year of first of 3 or more transplants	______________________

Source of first of 3 or more transplants	◯ donor brain death<br>◯ donor cardiac death<br>◯ living related donor<br>◯ living non related donor
Reason for failure of first of 3 or more transplants	___________________________________
Years of other transplants where 4 or more	____________________________
Source of other transplants where 4 or more and reason for failure	___________________________________

S4 KidneyOme Gene List

ACE	C5orf42	COQ6	FAN1	IFT57	NEK1	SBDS	TMEM107
ACTN4	CA2	COQ7	FAT1	IFT80	NEK8	SCARB2	TMEM138
ACVR2B	CASR	COQ8B	FGF10	IFT81	NIPBL	SCL7A7	TMEM216
ADAMTS13	CC2D2A	COQ9	FGF23	INF2	NME8	SCLT1	TMEM231
ADCK4	CCDC103	CRB2	FGF8	INPP5E	NODAL	SCNN1A	TMEM237
ADCY10	CCDC114	CRELD1	FGFR1	INVS	NOTCH2	SCNN1B	TMEM67
AGT	CCDC151	CSPP1	FGFR2	IQCB1	NPHP1	SCNN1G	TNXB
AGTR1	CCDC28B	CTNS	FN1	ITGA3	NPHP3	SDCCAG8	TRAF3IP1
AGXT	CCDC39	CUBN	FOXC1	ITGA8	NPHP4	SEC61A1	TRAP1
AHI1	CCDC40	CUL3	FOXC2	ITGB4	NPHS1	SEC63	TRIM32
ALMS1	CCDC41	CYP11B2	FRAS1	JAG1	NPHS2	SEMA3A	TRPC6
ALPL	CCDC65	CYP24A1	FREM1	KAL1	NR3C2	SGPL1	TRPM6
ANKS6	CCNO	CYP27B1	FREM2	KCNA1	NUP107	SIX1	TSC1
ANLN	CD151	CYP2R1	FXYD2	KCNJ1	NUP93	SIX2	TSC2
ANOS1	CD2AP	DCDC2	GALNT3	KCNJ10	OCRL	SIX5	TTC21B
AP2S1	CD46	DDX59	GANAB	KCNJ5	OFD1	SLC12A1	TTC8
APOL1	CDC73	DGKE	GAS8	KDM6A	PAX2	SLC12A3	TXNDC15
APRT	CENPF	DHCR7	GATA3	KIAA0556	PBX1	SLC16A12	UMOD
AQP2	CEP104	DMP1	GDF1	KIAA0586	PCBD1	SLC22A12	UPK3A
ARHGAP24	CEP120	DNAAF1	GLA	KIAA0753	PDE6D	SLC26A1	VDR
ARHGDIA	CEP164	DNAAF2	GLI3	KIF14	PDSS2	SLC2A2	VIPAS39
ARL13B	CEP290	DNAAF3	GLIS2	KIF7	PHEX	SLC2A9	VPS33B
ARL6	CEP41	DNAAF5	GNA11	KL	PKD1	SLC34A1	WDPCP
ARMC4	CEP83	DNAH11	GPC3	KLHL3	PKD2	SLC34A3	WDR19
ATP6V0A4	CFAP53	DNAH5	GRHPR	KMT2D	PKHD1	SLC3A1	WDR34
ATP6V1B1	CFB	DNAI1	GRIP1	KYNU	PLCE1	SLC41A1	WDR35
AVP	CFC1	DNAI2	GSN	LAMB2	POC1B	SLC4A1	WDR60
AVPR2	CFH	DNAL1	HAAO	LBR	PRKCSH	SLC4A4	WDR73
B9D1	CFHR1	DRC1	HNF1B	LCAT	PTH1R	SLC7A9	WNK1
B9D2	CFHR3	DSTYK	HNF4A	LMNA	PTHB1	SLC9A3R1	WNK4
BBIP1	CFHR5	DYNC2H1	HOGA1	LMX1B	PTPRO	SLIT2	WNT4
BBS1	CFI	DYX1C1	HOXA13	LRIG2	REN	SMARCAL1	WT1
BBS10	CHD1L	DZIP1L	HOXA4	LRP4	RET	SOX17	XDH
BBS12	CHD7	EHHADH	HOXB6	LRRC6	RMND1	SPAG1	XPNPEP3
BBS2	CLCN5	EMP2	HPRT1	LYZ	ROBO2	SRGAP1	ZIC3
BBS4	CLCNKA	ENPP1	HPSE2	LZTFL1	ROR2	STX16	ZMYND10
BBS5	CLCNKB	EVC	HSD11B2	MAGED2	RPGR	TAPT1	ZNF423
BBS7	CLDN16	EVC2	HYDIN	MAGT1	RPGRIP1L	TBC1D1	
BBS9	CLDN19	EXOC3L2	HYLS1	MAPKBP1	RRM2B	TBC1D32	
BICC1	CNNM2	EYA1	IFT122	MCIDAS	RSPH1	TBX18	
BMP4	COL4A1	EZH2	IFT140	MKKS	RSPH4A	TCTN1	
BSND	COL4A3	FAH	IFT172	MKS1	RSPH9	TCTN2	
C21orf59	COL4A4	FAM111A	IFT27	MUC1	SALL1	TCTN3	
C2CD3	COL4A5	FAM20A	IFT43	MYH9	SALL4	TFAP2A	
C3	COQ2	FAM58A	IFT52	MYO1E	SARS2	THBD	

S5 Table HIDDEN inclusion and exclusion criteria

Inclusion criteria	*Exclusion Criteria*
• Absence of identified cause or aetiology for kidney disease<br>• CKD Stage 5 (eGFR ≤ 15ml/min/1.73m^2; CKD-EPI equation) at ≤ 50 years of age<br>• Negative genetic test if a specific genetic kidney diagnosis has been suspected	• Likely or proven diabetic nephropathy, renovascular disease, renal sarcoidosis, primary nephrotic-range proteinuric disorder, or tuberculosis<br>• Paraproteinemia (except when excluded on kidney biopsy), exposure to nephrotoxin causing kidney dysfunction, or obstructive uropathy<br>• Nephromegaly (>14 cms for adults; normagram for paediatric patients) and a family history of cystic kidneys.<br>• Isolated Congenital Anomaly of Kidney and Urinary Tract (CAKUT)<br>• Identified glomerular disorder on kidney biopsy that clarifies a diagnosis<br>• Identified and proven primary renal diagnosis (as per ANZDATA coding)

S6 Table Whole exome sequencing studies in CKD patients of unknown aetiology					
**Study**	**Aim**	**Population**	**Diagnostic yield**	**Knowledge gap**	**Learning points**
Connaughton et al 2019[20]	To determine the likelihood of detecting monogenic causes of CKD in adults using whole exome sequencing (WES)	Cohort of 114 families including 138 affected individuals with CKD presenting in multicentre nephrology services	56%	51% no molecular genetic diagnosis was established with WES. 15% of monogenic causes of disease are complex deletion-insertion, copy-number variants, or reside within a promotor or other intronic region.	Complex deletion-insertion, copy-number variants, or reside within a promotor or other intronic region can not be detected by WES. This may explain why 51% remain without a molecular diagnosis.
Ottlewski et al 2019[18]	To systematically assess patients on the kidney transplant waitlist for their pre summed [illegible]etiologies using WES.	Single-centre cohort study of 57 CKD patients on transplant waitlist	12%	Targeted panel of 209 genes comprised a large number of CKD-associated genes, various genes were not covered because of technical limitations of the applied panel	Complete coverage of a growing number of kidney disease genes is warranted by whole exome or genome sequencing instead of targeted NGS approaches.
Lata et al 2018[21]	To study the diagnostic utility of WES in a selected referral population of adults with CKD.	Observational cohort of 92 patients from a single site major academic medical centre	56%	WES does not provide uniform coverage of all coding segments of the genome and may fail to capture some diagnostically relevant genomic regions, such as genomic imbalances and mutations in noncoding regions of the genome	WES analysis of larger, unselected CKD cohorts will provide a better assessment of its overall diagnostic yield in nephrology practice.

Groopman et al 2018[2]	To conducted exome sequencing in two independent broader cohorts of patients with chronic kidney disease	A clinical trial of 3315 from 280 medical centres in 25 nations.	12%	Study underestimates the overall burden of genetic disorders among patients with nephropathy.	Limitations of exome sequencing include suboptimal coverage of some clinically relevant regions, such as the mitochondrial genome or the duplicated regions of PKD1 and detection of intronic and copy-number variants
